# Knowledge of disease, diagnosis, adherence and impact of research in an Irish cohort of patients with inflammatory arthritis

**DOI:** 10.12688/hrbopenres.13274.1

**Published:** 2021-05-28

**Authors:** Viviana Marzaioli, Mary Canavan, Alex Donnelly, Siobhan Wade, Alexander Fraser, Tim O'Sullivan, Sinead Harney, Arthritis Ireland, Douglas J. Veale, Ursula Fearon

**Affiliations:** 1 Molecular Rheumatology, Trinity College Dublin, Dublin, Ireland; 2 EULAR Centre of Excellence, Centre for Arthritis and Rheumatic Diseases, St Vincent's University Hospital, Dublin, Dublin, Ireland; 3 Patient Advocate, Dublin, Ireland; 4 Dept of Rheumatology, University of Limerick, Limerick, Ireland; 5 Rheumatology Dept, Cork University Hospital, Cork, Ireland; 6 Arthritis Ireland, Dubln, Ireland

**Keywords:** Inflammatory Arthritis, Patient Perspective, Adherence, Pregnancy

## Abstract

**Background:** Patient engagement with clinicians results in shared decision making and increased adherence to medication. However, in order for strong patient: clinician partnerships to be achieved, communication barriers need to be identified. Therefore, the aim of this study was to examine the level of understanding of inflammatory arthritis patients and the need for strong patient-partnership in research.

**Methods**: An online anonymous survey was distributed to patients living with inflammatory arthritis which addressed questions about diagnosis, routine tests, medications and how they work, medication adherence, disease flare, heredity, pregnancy, and patient involvement in research.

**Results: **There were 1,873 respondents, 1416 of which had inflammatory arthritis (IA)- rheumatoid arthritis (RA) (65.8%) and psoriatic arthritis (PsA) (34.2%). They were predominantly female (RA 86%, PsA 85 %), aged 55±13 and 50±12 years. Less than 35% of patients had an understanding of diagnostic tests, what was measured and the implication for disease, with 75.5% also concerned about heredity. There was a high level of understanding of how specific medications treat inflammatory arthritis (72.9%). Adherence was also very high (>87%), with the main reasons for stopping medication without the advice of their clinician,  ‘feeling better’ and ‘side effects’ however  a significant proportion of patients (69.9%) reported a disease-flare following cessation of medication. Patients (31%) were also concerned that inflammatory arthritis reduced their chances of getting pregnant, with only 8% believing arthritis medications were safe to take during pregnancy. Finally, only 9% of patients had ever been asked to participate in a research study.

**Conclusions:** This study demonstrates a need for the development of stronger patient-partnerships with clinicians and researchers in relation to patient education and engagement with research, to create a platform where patients can have meaningful input and involvement in future research studies.

## Introduction

Arthritis is a leading cause of joint deformity and disability that affects 15% of the population, 2% of which suffer from inflammatory arthritis (IA) including rheumatoid arthritis (RA) and psoriatic arthritis (PsA) ^1– 3^. IA is an important chronic rheumatic and musculoskeletal disease (RMD) worldwide, causing significant morbidity, disability and increased mortality. It also reduces mobility and quality-of-life (QOL) thereby increasing social isolation and is associated with significant comorbidities ^4^. The costs of IA to both the individual and society are high, including economic and social costs, drugs, hospitalizations, lost workdays, cost to family and carers and an overall reduced QOL ^5^.

Targeted biotherapeutics have significantly improved outcomes for patients with IA particularly in early disease ^1– 3^. Indeed studies have shown that early intervention improves long-term outcomes, with data from inception longitudinal cohorts showing that effective treatment within the first 12 months of diagnosis is associated with better outcomes after 12-years of follow-up ^6^. Still however a significant proportion of patients have sub-optimal responses, associated adverse events or no response ^1– 3^. Currently, it is difficult to predict who will develop severe, erosive disease or who will respond to treatment. This is due to the complex underlying mechanisms of disease associated with disease pathogenesis in patients with IA, but also due to a gap in patient education and engagement, particularly with regard to their understanding of their disease, their diagnostic tests, their treatment strategy and adherence to medication ^7^. Indeed adherence to medication has been shown to be variable in patients with IA ranging from 50 to 80%, an effect that subsequently can significantly impact response rates ^3, 4, 6– 8^. It is clear that medication cannot be effective in a patient if it is not taken, however several studies have recognised non-adherence in patients with IA ^9^. 

Both clinical, translational, and indeed preclinical research are fundamental to the better understanding of disease diagnoses, progression and response which in turn will lead to innovative healthcare and possibly a more personalised medicine approach. However, for this to succeed, meaningful involvement of patients is central to the process. Thus, patient education is vital in this area to facilitate understanding of their disease, but also how their engagement with preclinical researchers will fundamentally impact on study-design, data interpretation and ultimately outcomes. However, such meaningful engagement is difficult due to lack of a structured environment for researchers (clinical and scientific) and patients to interact. Understanding these challenges and obstacles from both the patient’s and researcher’s perspective is critical in the development of better well defined pre-clinical studies that involve patient input. 

In this study we analysed from the patient’s perspective their understanding of their disease, the challenges and concerns they have with regard to their diagnoses. Therefore, in a collaborative approach involving clinicians, translational scientists, patient partner representative, and the patient advocate group Arthritis Ireland we identified conceptually important and relevant constructs for survey question generation which were incorporated in the ‘patients awareness survey’, to specifically address patient understanding of their diagnosis, diagnostic tests, medication and how they work, adherence and disease flare, issues of heredity and pregnancy, and patient involvement in research. This survey was utilised to develop an innovative approach to engage patients through the development of national ‘patient education awareness workshops’, with meaningful engagement between the three partners, the patient, the clinical and the scientist. In parallel, further patient’s feedback to specific discussion topics within the workshops was assessed which will be utilised to facilitate further refinement of patient engagement activities at the earliest stage of diagnoses where a coordinated approach to formulation of the idea with regard to patient education and engagement with research maintained into the future thereby nurturing the development of a robust process of patient partnership with researchers. 

## Methods

### Study design

This was a cross-sectional observational study. The survey was available on the Arthritis Ireland website and the social media platforms, Twitter and Facebook from 9 ^th^ April 2020 to 17 ^th^ May 2020.

### Information to participants before survey commencement

The survey was only intended for people living with IA, the aim of which was to identify participants’ understanding of their disease. If participants were unsure if they had an inflammatory form of arthritis or had any concerns regarding any questions outlined in the survey, information at the beginning of the survey directed them to visit the Arthritis Ireland Website for further clarity, where information and booklets on all types of arthritis, treatment for arthritis, pregnancy and information booklets are all clearly outlined.

### Survey

The objective of the survey was to assess patients understanding of their diagnosis, routine tests, medications and how they work, medication adherence, disease flare, heredity, pregnancy, and patient involvement in research. Demographics (age and gender) and clinical diagnoses were collected on all participants, in addition to specific survey questions. The study ran for approximately 6 weeks online to ensure the maximum amount of reach and participation. Consent was obtained upon initial commencement of the survey. 

To define the questions included in the survey we performed an ace validity test. This involved a literature review to define and conceptualise important and relevant constructs for survey item generation which included input from a multidisciplinary team of clinicians, scientific researchers, patient partner and patient advocate group Arthritis Ireland. Based on ace validity testing a consensus on questions to be inputted into the survey was agreed by the multidisciplinary team. The anonymous survey addressed patient understanding of their diagnosis, diagnostic tests performed at outpatient clinics, their treatments and how they worked. Questions also addressed adherence and disease flare, issues of heredity and pregnancy, and patient involvement in research. The survey can be found as extended data ^10^.

This study focused specifically on IA patients however responses from patients with diseases other than IA which encompassed gout, ankylosing spondylitis and juvenile idiopathic arthritis are currently being used for subsequent analysis and research. Moreover, responses to questions regarding exercise will also be used within the context of another study.

### Ethical approval

Ethical approval (No. 20191203) was granted by School of Medicine Research Ethics Committee, Trinity College Dublin.

### Statistical analysis

Data are presented as percentages and stratified between male/female or RA/PsA where required. Differences between groups were assessed using Chi square test with a confidence interval of 95%. GraphPad Prism 7.0 was used for the analyses. R can be used as an open source alternative.

## Results

### Participant demographics

The survey was completed by 1,873 patients, with 932 with RA (49.8%), 485 PsA (25.9%), 161 ankylosing spondylitis (8.6%), 39 juvenile idiopathic arthritis (2.1%), 61 gout (3.2%) or 195 other (10.4%) ^11^. For the purpose of this study we focused on patients with the two most common forms of IA (1416 patients), of which 65.8% had RA 34.2% had PsA. Table 1 illustrates demographics of the participants. Respondents were predominantly female (1210, 85.5% F vs 206, 14.5% M), with a F:M ratio of 6:1 (p< 0.05) in RA and 5.6:1 (p< 0.05) in PsA. Median age was 55±13 years for participants with RA and 50±12 years for participants with PsA. Participants were on a range of medications from basic pain relief to biologic agents. 

**Table 1.** Participants demographics. *p<0.05 Chi-Square test between Female vs Male percentages.

	RA	PsA
**AGE**	55±13	50±12
**Gender (F:M)**	6: 1 (798:133)	5.6: 1 (412:73)
**Medications**		
Pain Relievers	27.0% (251)	24.3% (118)
DMARDs	21.8% (203)	18% (87)
NSAIDS	16.0% (149)	16% (78)
Steroids	8.4% (78)	7% (34)
Biologics	17.8% (165)	26% (126)
Biosimilars	2.4% (22)	3% (14)
Methotrexate	6.7% (63)	6% (28)

### Patient responses to knowledge of disease activity measures

**Figure 1.**
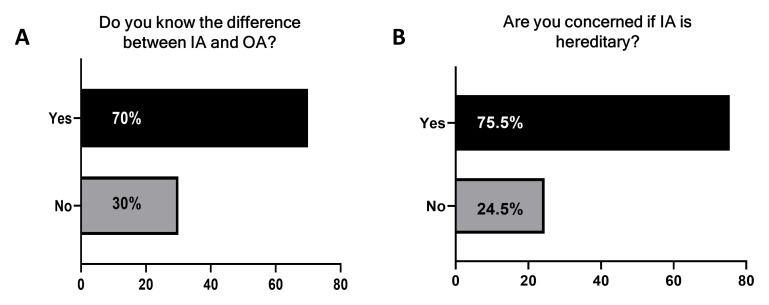
Participants’ understanding of inflammatory arthritis (IA) and its heritability. Participants were asked **A**) if they knew the difference between IA and osteoarthritis (OA) and **B**) if they were concerned IA was hereditary. Yes (black) and No (grey).

**Figure 2.**
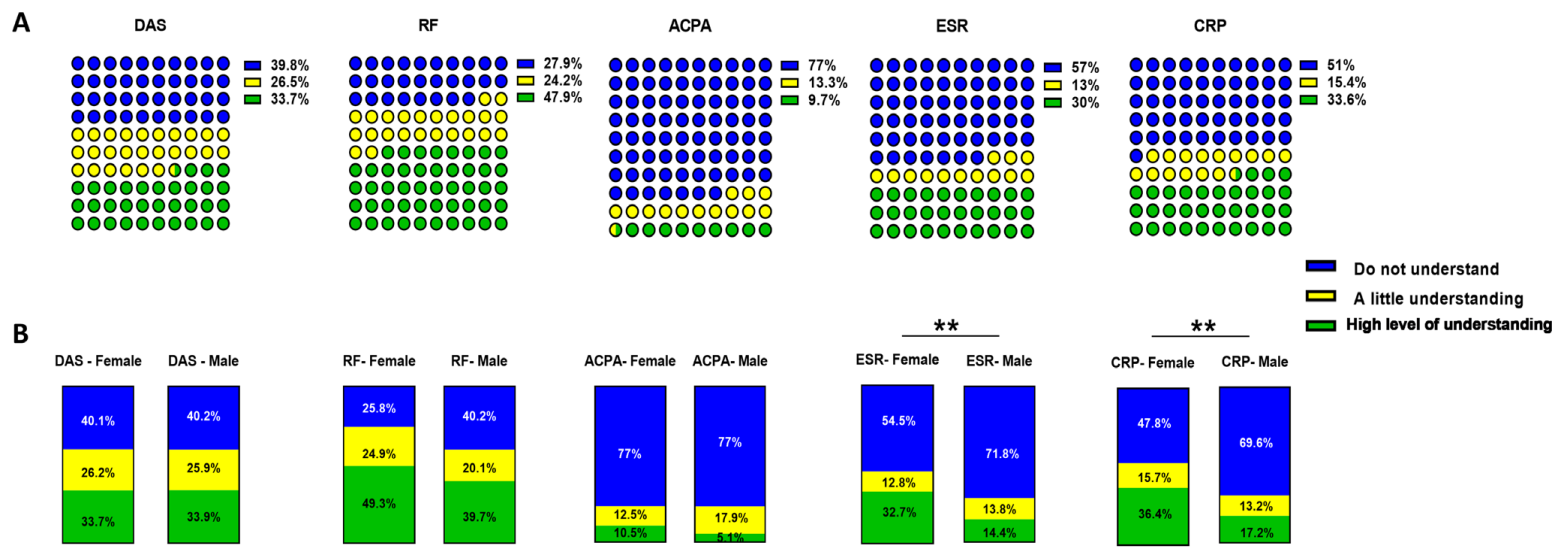
Participants' understanding of inflammatory arthritis (IA). DAS, RF, ACPA, ESR and CRP diagnostic test understanding among the **A**) IA respondents and **B**) stratified between female and male participants. Patients indicate their understanding of parameters on a Scale 1–10 (1=don’t understand at all, 10=understand very clearly). In the latter case answers were clustered the data as follow: 1–3 Do not understand (in blue), 4–6 A little understanding (in yellow), 7–10 High level of understanding (in green). Differences between female and male groups were assessed using Chi square test with a confidence interval of 95%. ** p<0.01.

Most patients understood the difference between IA and osteoarthritis (991 participants, 70% vs 425 participants, 30%; Figure 1A). In total, 75.5% (1069 participants) were concerned IA was hereditary ( Figure 1B), which was similar between RA and PsA patients, as well as between female and male participants (data not shown). Patients were asked to indicate their understanding of diagnostics tests performed at out-patient clinics (Scale 1–10; 1=don’t understand at all, 10=understand very clearly. Responses were subsequently stratified as “no understanding” 1–3, “little understanding” 4–6 and “high level of understanding” 7–10). For all diagnostic tests fewer than 35% of patients had a high level of understanding of what was measured and the implication for disease on day of clinic visit, these included C-reactive protein (CRP), erythrocyte sedimentation rate (ESR), anti-citrullinated protein antibodies (ACPA), visual analog score (VAS) and disease activity score 28 (DAS28), with a higher understanding only of the RF (678 participants, 47.9%) ( Figure 2A), with less than 10% understanding the ACPA test. There were no significant differences in understanding diagnostic test between RA and PsA participants (not shown), however when comparing the female and male participants, both CRP and ESR showed higher level of understanding by female participants ( Figure 2B, CRP 367/1008 participants, 36.4% vs 30/174 participants, 17.2%, p<0.01; ESR 330/1008 participants, 32.7% vs 25/174 participants, 14.4%, p<0.01), with similar understanding across groups for ACPA and DAS28 and RF ( Figure 2B).

### Patient understanding of how their medication works

**Figure 3.**
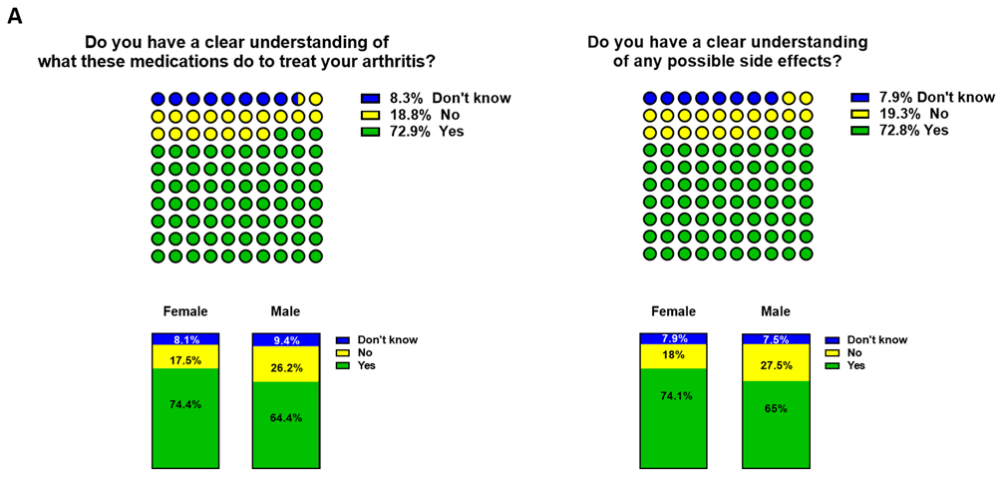
Participants' understanding of inflammatory arthritis (IA) medication. Participants were asked **A**) whether they had a clear understanding of IA medication and **B**) possible side effects. In blue ‘don’t know’, in yellow ‘No’ and green ‘Yes. Top general IA respondents, bottom female vs male participants.

In total, 829 participants (72.9%) understood how specific medications treat IA, however still a significant proportion of patients (18.8%, 213) had no understanding ( Figure 3A). No differences were observed between RA and PsA and female vs male. Similarly, the vast majority of participants (826, 72.8%) stated that they were aware of possible side effects of medications, with 19.3 % (219) unclear of medication side effects ( Figure 3B). This was not different among RA vs PsA or gender.

### Patient adherence to medication

**Figure 4.**
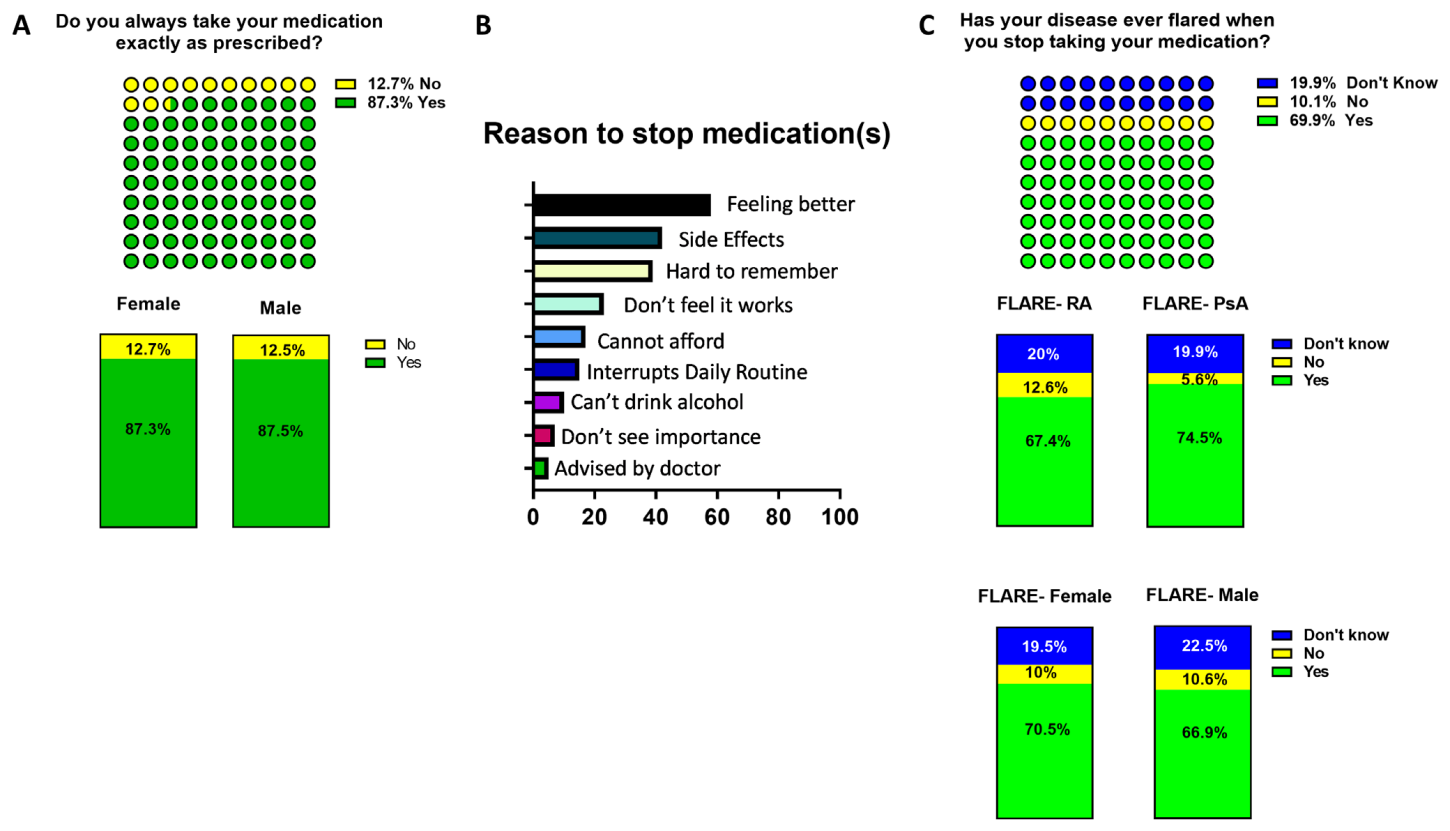
Adherence to inflammatory arthritis (IA) medication and flare. Participants were asked **A**) their adherence to medication (yellow ‘No’, green ‘Yes’) and **C**) disease flare occurrence in case they stopped their medication (blue ‘don’t know’, in yellow ‘No’ and green ‘Yes’). Top total IA respondents, bottom female vs male participants and RA vs PsA. The reasons for stopping medication are indicated in **4B**. The participants which declared to have stopped medication without been advised by their doctor were asked the reason for not adhering to medications. Reasons are listed as percentages.

Interestingly when patients were asked in this study ‘Do you always take your medication exactly as prescribed, other than to be told to stop by your doctor?’ adherence was very strong with >87% vs 12% (990 vs 144 participants) adhering to their medication ( Figure 4A). Adherence to medication was similar amongst female and male participants (87.3% v 87.5%, 850 vs 140) and also between RA and PsA (not shown). Importantly, 69.9% of those patients (793) who stated that they did not adhere to their medication reported disease flare following cessation of medication ( Figure 4C). The proportion of patients who reported disease flares upon cessation of medication was higher in PsA compared to RA (74.5% v 67.45%, 306/410 vs 487/722 patients) ( Figure 4C). Of those stopping medication, without the advice of their doctor, the main reasons were ‘feeling better’ (58%, 821 participants), ‘hard to remember to take’ (39%, 552 participants) and ‘side effects’ (42%, 595 participants) ( Figure 4B). Although a proportion of patients also said that they could not afford their medication and this was their reason for stopping (17%, 241 participants) ( Figure 4B) this represents a significant number of biologic-treated patients ineligible for free medications, who cannot afford them. Altogether, this strongly emphasises the need for patients to strictly adhere to their prescribed medication to maintain disease control.

### Pregnancy and medication

**Figure 5.**
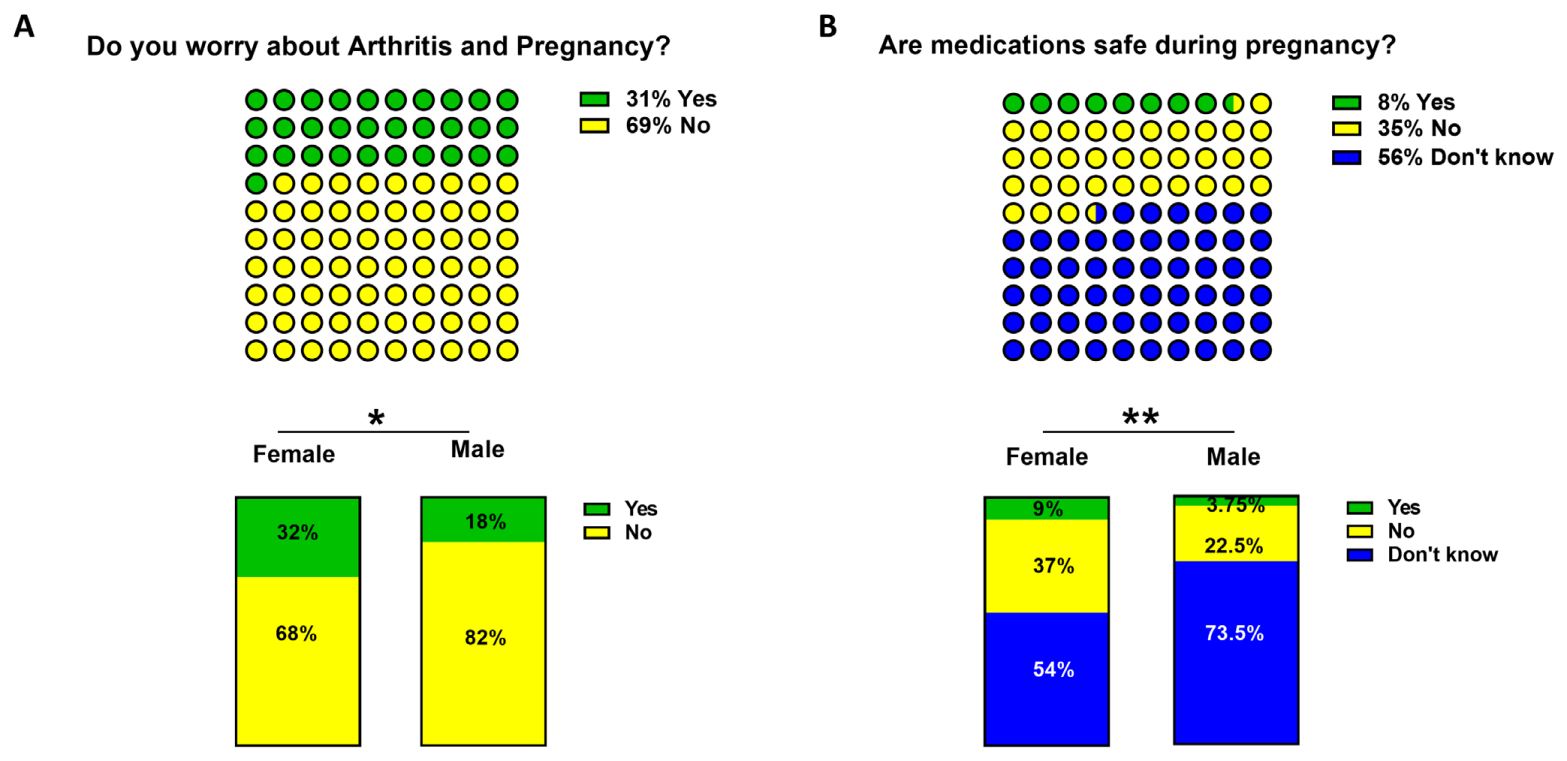
Inflammatory arthritis (IA) and pregnancy. Participants were asked **A**) whether they were worried about arthritis and pregnancy (yellow ‘No’, green ‘Yes’) and **B**) if medication were safe during pregnancy (blue ‘don’t know’, in yellow ‘No’ and green ‘Yes’). Differences between female and male groups were assessed using Chi square test with a confidence interval of 95%. *p< 0.05 and ** p<0.01.

Patients understanding of IA and pregnancy was identified as a potential significant unmet need. Although differences in patient education between centres was not captured, this data would highlight there is a need to standardise patient education at all centres nationally. Concern that IA would reduce their chances of getting pregnant was reported by 31% (130 participants) of respondents ( Figure 5A), as expected females were significantly more concerned about pregnancy related issues than males (32% v 18% p<0.05, 125 vs 5 participants). Perhaps of most importance is that the vast majority of respondents either did not know (56%, 638 participants) or believed that arthritis medications were not safe to take during pregnancy (35%, 400 participants) ( Figure 5B). Therefore, only 8% of respondents (94 participants) affirmed knowledge of the safety of arthritis medications during pregnancy, this is a highly significant gap in knowledge in a group of patients who are predominantly female and of childbearing age. Interestingly, a higher proportion of females *vs* males answered definitively ‘No’ regarding whether arthritis medication was safe to take during pregnancy (37% v 22.5%; p<0.05, 364/972 vs 36/161 participants), whereas more males answered, ‘don’t know’ (73.5% v 54%, 118/161 vs 520/972 p<0.01, Figure 5B).

### Patients’ involvement in research

**Figure 6.**
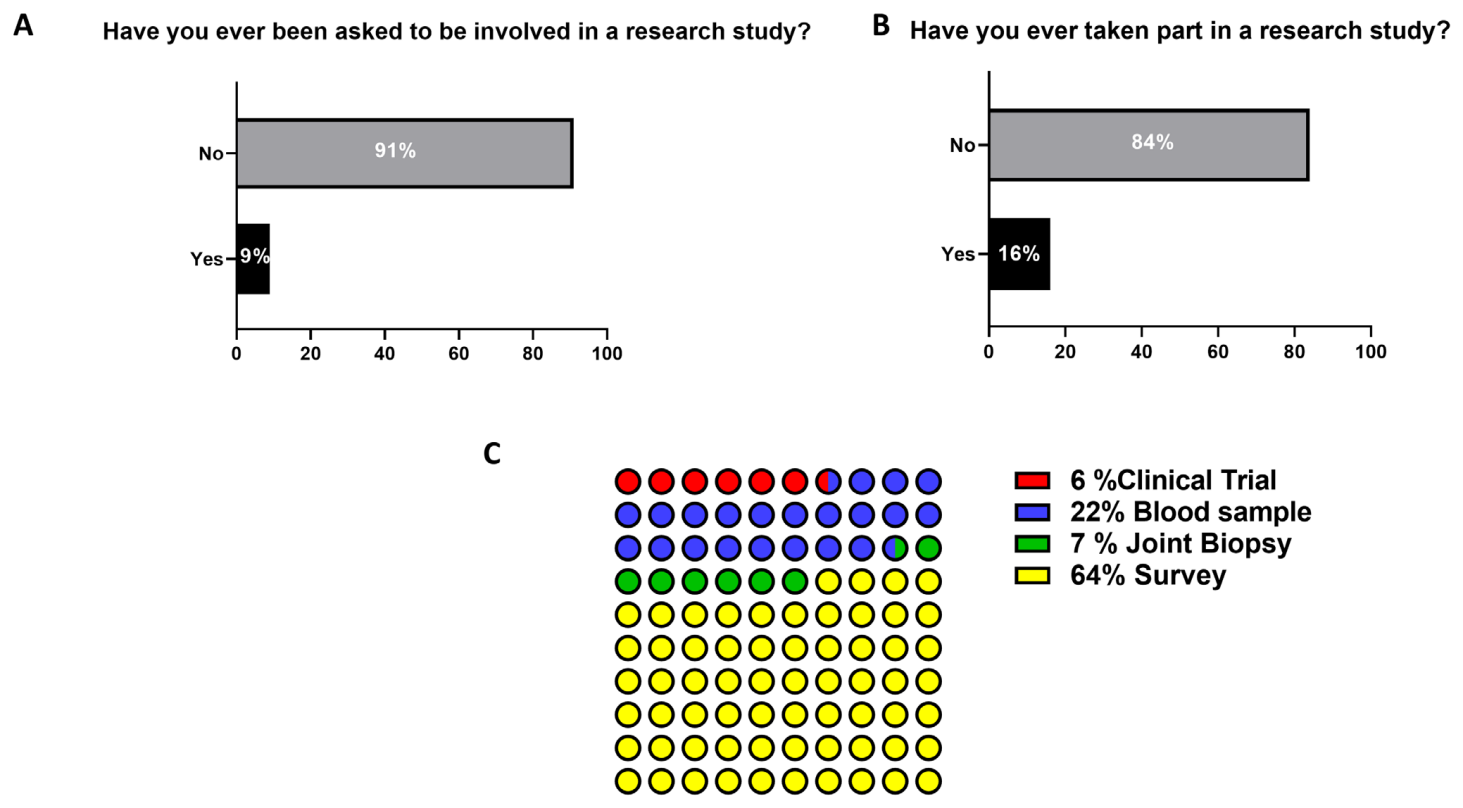
Participants’ involvement in research studies. Participants were asked **A**) whether they were asked in research studies and **B**) whether they took part in a research study. The participants which responded positively to the latter, where asked **C**) which kind of research study they were involved in.

For pre-clinical/translational research to have impact for disease outcomes, patient’s involvement in a meaningful way is critical for its success, thus patient education and engagement is vital. However, in this survey when questioned if patients had ever been asked to be involved in a research study, only 9% (127) of respondents answered yes ( Figure 6A), with only 16% of these participating (227) in a research study ( Figure 6B). Of those who had taken part in a research study the predominant type of research was survey (64%, 139 participants), blood donation (22%, 48 participants), arthroscopy (7%, 16 participants), and clinical trials testing new medications (6%, 14 participants) ( Figure 6C).

## Discussion

The objective of this study was to assess the understanding patients with IA have of their own diagnosis, routine diagnostics tests used in the clinical setting, medication adherence, disease flare, pregnancy and finally to ascertain their attitudes to research studies. The results highlight several patient concerns regarding their disease while also emphasising the need for increased patient education and engagement to improve patients understanding of their disease and develop strong, long-term patient-clinical-researcher partnerships, so that patients can contribute to future project ideas, development, and dissemination.

In this study while a significant proportion of patients understood the difference between IA and OA, a large proportion of respondents expressed concern that IA is a hereditary disease. While there is a genetic component to both RA and PsA, it is now widely accepted that it is a combination of genetic, environmental, and autoimmune factors that contribute to the disease pathogenesis ^12– 15^. In addition, while many genes have been identified in IA, their role in the pathogenesis remains poorly understood, with the exception perhaps of the human leukocyte antigen (HLA). The class II HLA DRB1*0404 allele has been identified as the strongest susceptibility factor for RA ^1, 3, 9, 12, 15^, while PsA is most commonly associated with the HLA class I genes HLA-B*27, HLA-C*06, HLA-C*07 and MICA-A9 conferring the strongest predisposition to PsA ^1, 3, 15– 17^. Previous studies measuring the degree of heritability of RA and PsA have produced widely different results, depending on the methods used, with >100 loci now associated with IA. One UK study which measured disease discordance in monozygotic and dizygotic twins estimated the disease heritability of RA to be around 60% ^18^, while similar studies in Denmark reported disease heritability of approximately 12% ^9^. In contrast, the heritability estimated from epidemiological studies in PsA has not been validated from twin studies, as no large PsA twin studies exist to date ^19^. The large degree in variability in these studies emphasises that a combination of multiple factors, in addition to genetics, are important in the pathogenesis of IA. Further clarification with patients upon diagnosis regarding these factors and how they can all contribute to the development of IA, may alleviate some concerns about hereditary issues.

A number of tests are utilised in the clinic to aid diagnosis, treatment and follow up of patients with IA. These tests help clinicians to establish a diagnosis but may also be useful to monitor response to treatment and predict disease severity. In this study, we establish that a significant number of patients had little or no understanding of these diagnostic tests, specifically ESR, CRP, RF, ACPA, and the composite disease activity score DAS28. Of note, ACPA was the diagnostic test which patients least understood. Approximately 70% of RA patients test positive for ACPA, with the majority of PsA negative. Studies have shown that ACPA ^+^ RA patients develop more erosive disease than ACPA ^-^ patients but they appear to have a better response to therapy ^20^. These tests are pivotal, not alone in making a diagnosis but also understanding the potential course of a patient’s disease, thus enabling clinicians to make decisions regarding treatment. The results of this survey emphasize the need to improve awareness among patients of these possible diagnostic and prognostic tests in order to empower patients to engage in shared decision-making with their rheumatologists. Such patient empowerment is important for patient education, but also towards developing a more meaningful type of patient-clinician relationship whereby patient and doctor can engage in important decision-making as a team rather than as individuals. Several studies examining the impact of patient empowerment on patient outcomes have demonstrated higher levels of patient satisfaction and adherence to treatment regimens, in patients who were actively involved in the decision-making process with their clinician ^21, 22^.

Reassuringly, a significant proportion of respondents in this study indicated that they understood how their medications treat their arthritis and possible side effects that may occur, with <20% of patients unaware of how their medications work or the possible side effects. The development of side effects has been shown to negatively impact adherence to treatment regimens ^23^, therefore, lack of awareness of these potential side effects may lead to increased non-adherence. It is important, therefore that patients are fully aware of the potential side effects of their medications, before they commence treatment, so they can distinguish symptoms associated with side effects and those as a result of the disease itself. Preparedness and increased awareness may reduce anxiety and improve adherence and compliance in their treatment regime.

Interestingly and reassuringly, our patients reported a very high degree of adherence with their prescribed medications compared to those in previous reports in which adherence levels ranged from 50–75% ^7, 8, 18^. It is recognised that non-adherence to disease modifying anti-rheumatic drugs (DMARD) treatment is associated with higher disease activity in early RA patients ^24^. Moreover, a meta-analysis published by Li *et al.,* reporting on 1,963 RA patients demonstrated that DAS28 score was significantly lower in adherent patients compared to non-adherent subjects ^25^. There are far fewer studies investigating adherence to medication for PsA patients; however, in one retrospective study of 325 patients with PsA, it was reported 76% adherence rate for anti-tumour necrosis factor (TNF) inhibitors users ^26^. In addition, it was reported that nonadherence to medication was consistently associated with psychological factors in PsA ^27^. These studies suggest that patients who adhere to their medication have better disease control. This is also reflected in our study, whereby >60% of respondents who indicated that they do not always adhere to their treatment regime reported subsequent flare in their disease. Indeed, a previous study by Hill *et al.* reported that an introduction of a patient education programme, involving one to one patient education sessions significantly increased adherence to medication in RA patients ^28^. An increase in patient education regarding flares resulting from cessation of medication, and further clarification on potential side effects may reduce non-adherence further.

 It is well established that the prevalence of RA is significantly higher in females than males where the incidence is 2–3 times higher below the age of 50 ^29^, with PsA prevalence equally distributed between male and female, with males tending to have a higher psoriasis area and severity index (PASI) score ^28^. Consequently, issues regarding pregnancy and childbirth are important considerations for the majority of patients diagnosed with IA, who are females in their reproductive years. This is an issue of particular importance as highlighted in a recent review, following which we established a specific maternal medicine clinic focused patients with arthritis ^29^. In our study, 31% of respondents indicated concern about arthritis and pregnancy while a significant percentage of respondents (91%) also indicated that arthritis medications were not safe in pregnancy or they did not know. This represents an important unmet need in patient understanding of arthritis medications and pregnancy, which must be addressed by increased education. A study by Chakravarty *et al*., involving two online surveys for physicians and patients regarding family planning issues, reported that the majority of rheumatologists discussed conception and pregnancy with their female patients. In our experience, patients believed that the information received varied depending on the source and there was little consensus among specialists in relation to the effect of pregnancy on arthritis and of arthritis medication on pregnancy, so we developed a combined update ^30^. In addition, the British Society for Rheumatology has now produced their own guidelines on these issues ^31^. However, less than half of patients with rheumatic diseases and planning to get pregnant reported consulting their patient's treating general practitioner/gynaecologist about these topics. Moreover in one study, the majority of patients reported that their pregnancy related concerns were not adequately addressed during their medical appointments ^32^. The results of this survey highlight the safety concerns of medications used to treat arthritis during pregnancy. Rheumatologists should discuss these concerns with patients of childbearing age and address issues such as safety of medication preconception, during pregnancy and during breastfeeding, in addition to highlighting issues regarding disease control before, during and after pregnancy. Rheumatologists should consult the European League Against Rheumatology (EULAR) “points to consider” on the use of anti-rheumatic drugs before and during pregnancy and lactation, when consulting with female patients who are contemplating pregnancy ^33^.

An important aspect of this study assessed the degree of patient involvement in research studies. Our results demonstrate that very few patients have participated in research studies, and this aspect was also highlighted in the Q&A sessions during the national workshops. A small percentage of patients had participated in a study, but the vast majority had taken part in a survey while very few respondents had participated in a clinical trial or bio-sampling studies. Translational research studies are essential in identifying new therapeutic targets for the treatment of IA, improving patient outcomes, predicting response to treatment and predicting disease course. Therefore, patient participation in these studies is critical in helping to achieve these aims. Through the patient centred workshops completed during this study, it is clear that many patients are willing and keen to take part in such studies but need more information as to how and when to engage with the research team in this respect, and this engagement needs to take place at the development stage of the research. Indeed, patients highlighted the need for studies that address the link between lifestyle and level of inflammation, with a particular emphasis on sleep, diet, anxiety, and exercise. Therefore, we need a structured environment for researchers (clinical and scientific) and patients to interact at the pre-clinical stage of research studies to enable patient input. Furthermore, it is important that principal investigators actively recruiting patients for studies advertise to a wide variety of patient groups in clinical settings outside hospitals, such as general practice (GP) surgeries, advocacy groups and community health centres.

In conclusion, this study analysed the patient’s perspective of their disease, and the challenges and concerns they have with regard to their diagnoses. We used an approach of surveying patients and organizing educational workshops to ascertain and fill the knowledge gaps and thus engage with a wide patient audience. This unique collaborative approach involved rheumatologists, scientists, a patient partner, and the national patient advocate group Arthritis Ireland to identify conceptually important and relevant constructs to develop the ‘patients awareness survey’, specifically addressing items of understanding of diagnosis, diagnostic tests, medication and adherence, disease flare, heredity, pregnancy and patient involvement in research. This survey facilitated an innovative approach to engage patients through the development of a national network of ‘patient education and awareness workshops’, with meaningful engagement with the patients, the clinicians and the scientists. The results are informative, positively highlighting patients have a good understanding of their medication and adherence and are eager to engage in research studies. Importantly however, we also identified significant gaps in patient education, which highlighted poor understanding of diagnostic tests, concerns with regard to pregnancy and hereditary and lack of engagement in the development of research studies, these are important knowledge gaps that we must addressed in our practice plan.

## Data availability

### Underlying data

Figshare: Patient Awareness Survey Responses Molecular Rheumatology TBSI.xlsx https://doi.org/10.6084/m9.figshare.14414144 ^11^.

### Extended data

Figshare: Patient Awareness Survey Questions. https://doi.org/10.6084/m9.figshare.14573127 ^10^.

Data are available under the terms of the Creative Commons Attribution 4.0 International license (CC-BY 4.0).
