# Knowledge of disease, diagnosis, adherence and impact of research in an Irish cohort of patients with inflammatory arthritis

**DOI:** 10.12688/hrbopenres.13274.2

**Published:** 2023-04-27

**Authors:** Viviana Marzaioli, Mary Canavan, Alex Donnelly, Siobhan Wade, Alexander Fraser, Tim O'Sullivan, Sinead Harney, Arthritis Ireland, Douglas J. Veale, Ursula Fearon

**Affiliations:** 1 Molecular Rheumatology, Trinity College Dublin, Dublin, Ireland; 2 EULAR Centre of Excellence, Centre for Arthritis and Rheumatic Diseases, St Vincent's University Hospital, Dublin, Dublin, Ireland; 3 Patient Advocate, Dublin, Ireland; 4 Dept of Rheumatology, University of Limerick, Limerick, Ireland; 5 Rheumatology Dept, Cork University Hospital, Cork, Ireland; 6 Arthritis Ireland, Dubln, Ireland

**Keywords:** Inflammatory Arthritis, Patient Perspective, Adherence, Pregnancy

## Abstract

**Background:** Patient engagement with clinicians results in shared decision making and increased adherence to medication. However, in order for strong patient: clinician partnerships to be achieved, communication barriers need to be identified. Therefore, the aim of this study was to examine the level of understanding of inflammatory arthritis patients and the need for strong patient-partnership in research.

**Methods**: An online anonymous survey was distributed to patients living with inflammatory arthritis which addressed questions about diagnosis, routine tests, medications and how they work, medication adherence, disease flare, heredity, pregnancy, and patient involvement in research.

**Results: **There were 1,873 respondents, 1416 of which had inflammatory arthritis (IA)- rheumatoid arthritis (RA) (65.8%) and psoriatic arthritis (PsA) (34.2%). They were predominantly female (RA 86%, PsA 85 %), aged 55±13 and 50±12 years. Less than 35% of patients had an understanding of diagnostic tests, what was measured and the implication for disease, with 75.5% also concerned about heredity. There was a high level of understanding of how specific medications treat inflammatory arthritis (72.9%). Adherence was also very high (>87%), with the main reasons for stopping medication without the advice of their clinician,  ‘feeling better’ and ‘side effects’ however  a significant proportion of patients (69.9%) reported a disease-flare following cessation of medication. Patients (31%) were also concerned that inflammatory arthritis reduced their chances of getting pregnant, with only 8% believing arthritis medications were safe to take during pregnancy. Finally, only 9% of patients had ever been asked to participate in a research study.

**Conclusions:** This study demonstrates a need for the development of stronger patient-partnerships with clinicians and researchers in relation to patient education and engagement with research, to create a platform where patients can have meaningful input and involvement in future research studies.

## Significance and Innovations

- Unmet needs in patient knowledge include diagnostic tests, heredity, pregnancy and the impact of research.
- Patients had a good understanding of their diagnosis, medications and side effects, including the importance of adherence, however a significant percentage reported that stopping treatment without medical consultation resulted in a flare of the disease.
- The study highlights the importance of a better communication between patients and rheumatologists to explain diagnostic tests, effects of pregnancy and heredity, and the importance of engaging patients in research, especially in areas such as diet, stress and exercise.

## Introduction

Arthritis is a leading cause of joint deformity and disability that affects 15% of the population, 2% of which suffer from inflammatory arthritis (IA) including Rheumatoid Arthritis (RA) and Psoriatic Arthritis (PsA) ^1– 3^. IA is an important chronic RMD worldwide, causing significant morbidity, disability and increased mortality. It also reduces mobility and quality-of-life (QOL) thereby increasing social isolation and is associated with significant comorbidities ^4^. The costs of IA to both the individual and society are high, including economic and social costs, drugs, hospitalizations, lost workdays, cost to family and carers and an overall reduced QOL.

Targeted biotherapeutics have significantly improved outcomes for patients with IA particularly in early disease ^1– 3^. Indeed studies have shown that early intervention improves long-term outcomes, with data from inception longitudinal cohorts showing that effective treatment within the first 12 months of diagnosis is associated with better outcomes after 12-years of follow-up ^5^. Still however a significant proportion of patients have sub-optimal responses, associated adverse events or no response ^1– 3^. Currently, it is difficult to predict who will develop severe, erosive disease or who will respond to treatment. This is due to the complex underlying mechanisms of disease associated with disease pathogenesis in patients with IA, but also due to a ‘GAP’ in patient education and engagement, particularly with regard to their understanding of their disease, their diagnostic tests, their treatment strategy and adherence to medication. Indeed adherence to medication has been shown to be variable in patients with IA ranging from 50 to 80%, an effect that subsequently can significantly impact response rates ^3, 4, 6– 8^. It is clear that medication cannot be effective in a patient if it is not taken, however several studies have recognised non-adherence in patients with IA ^9^. 

Both clinical, translational, and indeed preclinical research are fundamental to the better understanding of disease diagnoses, progression and response which in turn will lead to innovative healthcare and possibly a more personalised medicine approach. However, for this to succeed, meaningful involvement of patients is central to the process. Thus, patient education is vital in this area to facilitate understanding of their disease, but also how their engagement with preclinical researchers will fundamentally impact on study-design, data interpretation and ultimately outcomes. However, such meaningful engagement is difficult due to lack of a structured environment for researchers (clinical and scientific) and patients to interact. Understanding these challenges and obstacles from both the patient’s and researcher’s perspective is critical in the development of better well defined pre-clinical studies that involve patient input. 

In this study we analysed from the patient’s perspective their understanding of their disease, the challenges and concerns they have with regard to their diagnoses. Therefore, in a collaborative approach involving clinicians, translational scientists, patient partner representative, and the patient advocate group Arthritis Ireland we identified conceptually important and relevant constructs for survey question generation which were incorporated in the ‘patients awareness survey’, to specifically address patient understanding of their diagnosis, diagnostic tests, medication and how they work, adherence and disease flare, issues of heredity and pregnancy, and patient involvement in research. This survey was utilised to develop an innovative approach to engage patients through the development of national ‘patient education awareness workshops’, with meaningful engagement between the three partners, the patient, the clinical and the scientist. In parallel, further patient’s feedback to specific discussion topics within the workshops was assessed which will be utilised to facilitate further refinement of patient engagement activities at the earliest stage of diagnoses where a coordinated approach to formulation of the idea with regard to patient education and engagement with research maintained into the future thereby nurturing the development of a robust process of patient partnership with researchers. 

## Methodology

### Study design

To define the questions included in the survey, a literature review and open discussion was used to define and conceptualise important and relevant constructs for survey item generation which included input from a multidisciplinary team of clinicians, scientific researchers, patient partner and patient advocate group Arthritis Ireland. This is a cross-sectional observational study. The anonymous survey addressed patient understanding of their diagnosis, diagnostic tests performed at outpatient clinics, their treatments and how they worked. Questions also addressed adherence and disease flare, issues of heredity and pregnancy, and patient involvement in research. The survey was only intended for people living with IA, the aim of which is to identify participants understanding of their disease. If participants were unsure if they had an inflammatory form of Arthritis or had any concerns regarding any questions outlined in the survey, information at the beginning of the survey directed them to visit the Arthritis Ireland Website for further clarity ( https://www.arthritisireland.ie; https://www.arthritisireland.ie/go/information), where information and booklets on all types of arthritis, treatment for arthritis, pregnancy and information booklets are all clearly outlined. Demographics (age and gender) and clinical diagnoses were collected on all participants, in addition specific survey questions. Questions were multi-choice with possibility with possibility of a single answer. Where question was not applicable to the participant N/A option was made available. The survey was completed by 1,873 patients, with RA (49.8%), PsA (25.9%), AnkSpon (8.6%), JIA (2.1%), Gout (3.2%) or other (10.4%). For the purpose of this study, we focused on patients with the two most common forms of IA (1416 patients), of which RA were 65.8% and PsA 34.2% (RA n=930. PsA n=485).

Following completion of surveys, round table discussions within a national workshop, focused on survey questions, with patient feedback that will be included to implement future workshops and PPI engagement. The survey was available via the Arthritis Ireland website and associated social media channels between 9 ^th^ April and 17 ^th^ May 2020.

### Ethical approval

Ethical approval was granted by School of Medicine Research Ethics Committee, Trinity College Dublin.

### Statistical analysis

Analysis was performed using GraphPad Prism 9. Data in graphs are presented as percentages and stratified between male/female or RA/PsA were required. Data are present as number of individuals in tables. Patients indicated their understanding of parameters on a Scale 1-10 (1=don’t understand at all, 10=understand very clearly). Answers were clustered as follow: 1-3 Do not understand, 4-6 A little understanding, 7-10 High level of understanding. Differences between groups were assessed on the number of individuals in contingency tables using Chi-square test with a confidence interval of 95%.

## Results

### Participant demographics

The survey was completed by 1,873 patients, with RA (49.8%), PsA (25.9%), AnkSpon (8.6%), JIA (2.1%), Gout (3.2%) or other (10.4%). For the purpose of this study, we focused on patients with the two most common forms of IA (1416 patients), of which RA were 65.8% and PsA 34.2%. Table 1 illustrates demographics of participants. Respondents were predominantly female (85.5% F vs 14.5%M), with F:M (6:1) in RA and (5.6:1) in PsA. Median age was 55±13 in RA and 50±12 in PsA. Participants were on a range of medications from basic pain relief to biologic agents ( Table 1).

**Table 1.** Participants demographics.

	RA	PsA
**AGE**	55±13	50±12
**Gender (F:M)**	6: 1 (798:133)	5.6: 1 (412:73)
**Medications**		
Pain Relievers	27.0% (251)	24.3% (118)
DMARDs	21.8% (203)	18% (87)
NSAIDS	16.0% (149)	16% (78)
Steroids	8.4% (78)	7% (34)
Biologics	17.8% (165)	26% (126)
Biosimilars	2.4% (22)	3% (14)
Methotrexate	6.7% (63)	6% (28)

### Diagnostic tests understanding

**Figure 1.**
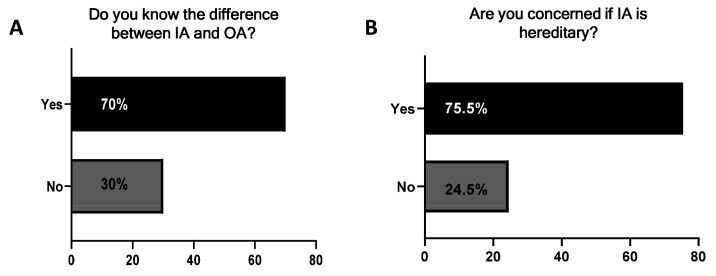
Participants’ understanding of inflammatory arthritis (IA) and its heritability. Participants were asked **A**) if they knew the difference between IA and osteoarthritis (OA) and **B**) if they were concerned IA was hereditary. Yes (black) and No (grey).

**Figure 2.**
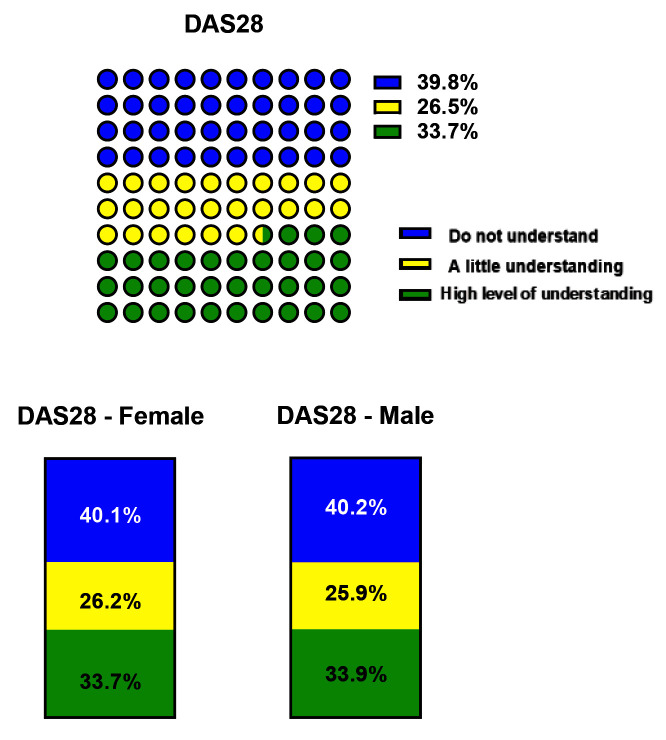
Participants' understanding of DAS28. **A**) IA respondents and **B**) stratified between female and male participants. Patients indicate their understanding of parameters on a Scale 1-10 (1=don't understand at all, 10=understand very clearly). In the latter case answers were clustered the data as follow: 1-3 Do not understand (in blue), 4-6 A little understanding (in yellow), 7-10 High level of understanding (in green). Differences among groups are represented in Table 2.

**Figure 3.**
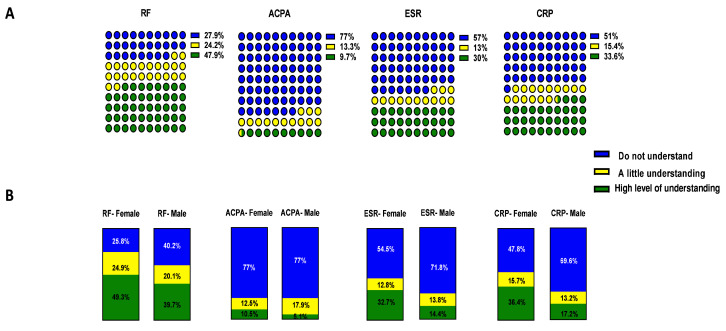
Participants' understanding of inflammatory arthritis (IA). RF, ACPA, ESR and CRP diagnostic test understanding among the **A**) IA respondents and **B**) stratified between female and male participants. Patients indicate their understanding of parameters on a Scale 1–10 (1=don’t understand at all, 10=understand very clearly). In the latter case answers were clustered the data as follow: 1–3 Do not understand (in blue), 4–6 A little understanding (in yellow), 7–10 High level of understanding (in green). ** p<0.01. Differences among groups are represented in Table 2.

**Figure 4.**
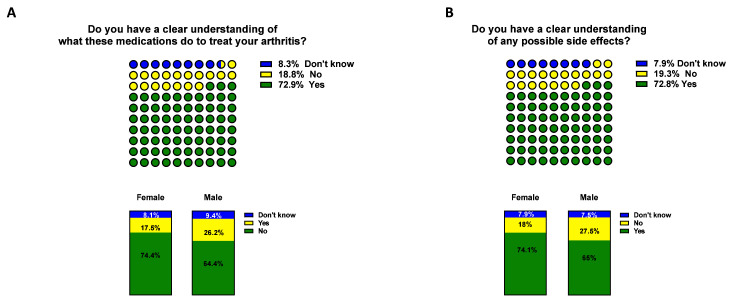
Participants' understanding of inflammatory arthritis (IA) medication. Participants were asked A) whether they had a clear understanding of IA medication and B) possible side effects. In blue 'don't know', in yellow 'No' and green 'Yes. Top, general IA respondents, bottom female vs male participants. Differences among groups are represented in Table 3..

Most patients understood the difference between IA and osteoarthritis (70% vs 30% Supplementary Figure 1A). 75.5% were concerned IA was hereditary ( Figure 1B), which was similar between RA and PsA patients, as well as between female and male participants (Data not shown). Patients were asked to indicate their understanding of diagnostics tests performed at out-patient clinics (Scale 1-10; 1=don’t understand at all, 10=understand very clearly). 39.85% of patients did not understand the measurement of disease activity (DAS28), although DAS28 is a more relevant parameter for disease activity in RA ^10^, no differences were observed between RA and PsA understanding ( Table 2); in addition, no gender differences were observed ( Figure 2 and Table 2). For all diagnostic tests less than 35% of patients had a high level of understanding of what was measured and the implication for disease on day of clinic visit, these included CRP, ESR, ACPA, VAS and, with a higher understanding only of the RF (47.9%) ( Figure 3A), with less 10% understanding the ACPA test. As expected, RA patients had a higher level of understanding of RF than PsA patients, (p< 0.01), being the factor more relevant in their disease; in addition, Female had a higher understanding of the disease than male (p<0.001) ( Figure 3B and Table 2). There were no significant differences in understanding diagnostic test between RA and PsA participants for ACPA, ESR and CRP ( Table 2), however when comparing the female and male participants, all showed higher level of understanding by female participants ( Figure 3B and Table 2, CRP 36.4% vs 17.2%, ESR 32.7% vs 14.4%, ACPA 10.5% vs 5.1%). Significantly, 72.9% of participants understood how specific medications treat IA, however still a significant proportion of patients 18.8% had no understanding ( Figure 4A). No differences were observed between RA and PsA, however female had a better understanding than male ( Table 3 p<0.01). Similarly, the vast majority of participants (72.8%) stated that they were aware of possible side effect of medications, with 19.3 % unclear ( Figure 4B). A trending difference between RA and PsA was observed (p=0.05) and a significant difference in gender (p<0.05, Table 3).

**Table 2.** Diagnostic test understanding absolute numbers.

Test	Answers	RA	PsA	p value	Female	Male	p value2
**DAS28**	Do not understand	300	174	*0.3575*	404	70	*0.515*
	A little understanding	191	118		246	45	
	High level of understanding	267	133		340	49	
**RF**	Do not understand	189	141	0.005**	260	70	0.0005***
	A little understanding	183	103		251	35	
	High level of understanding	386	181		497	69	
**ACPA**	Do not understand	570	341	*0.0948*	776	134	0.0234*
	A little understanding	105	52		126	31	
	High level of understanding	83	32		106	9	
**ESR**	Do not understand	439	236	*0.7163*	549	125	<0.0001****
	A little understanding	97	56		129	24	
	High level of understanding	222	133		330	25	
**CRP**	Do not understand	390	213	*0.6772*	482	121	<0.0001****
	A little understanding	112	71		159	23	
	High level of understanding	256	141		367	30

**Table 3.** Diagnostic test understanding absolute numbers.

Questions	Answers	RA	PsA	p value	Female	Male	p value2
**Do you have a clear understanding of what** **these medications do to treat your art**	Don't know	61	33	*0.6837*	79	15	0.0208*
	No	141	72		171	42	
	Yes	523	306		726	103	
**Do you have a clear understanding of any** **possible side effects?**	Don't know	59	30	*0.0556*	77	12	0.0177*
	No	154	65		175	44	
	Yes	510	316		722	104	
**Do you always take your medication** **exactly as prescribed?**	No	91	53	*0.8806*	124	20	*0.9352*
	Yes	632	358		850	140	
**Has your disease ever flared when you** **sbottom taking your medication?**	Don't know	144	82	0.0007***	190	36	*0.6294*
	No	91	23		97	17	
	Yes	487	306		686	107	
**Do you worry about arthritis and** **pregnancy?**	No	196	94	*0.0501*	267	23	*0.1208*
	Yes	75	55		125	5	
**Are arthritis medications safe during** **pregnancy?**	Don't know	403	235	*0.1862*	520	118	<0.0001****
	No	250	150		364	36	
	Yes	68	26		88	6	

### Adherence to medication

**Figure 5.**
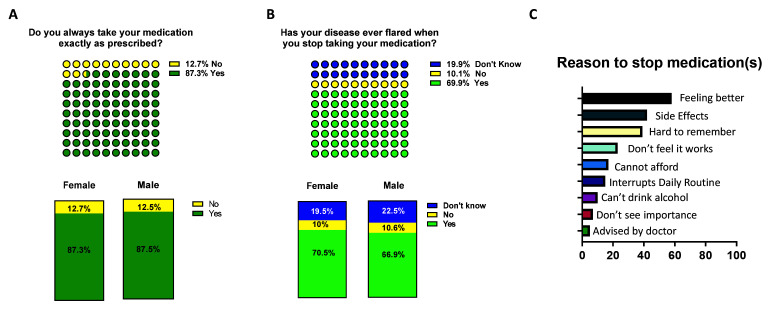
Adherence to inflammatory arthritis (IA) medication and flare. Participants were asked **A**) their adherence to medication (yellow 'No', green 'Yes') and **B**) disease flare occurrence in case they stopped their medication (blue 'don't know', in yellow 'No' and green 'Yes'). Top, total IA respondents, bottom female vs male participants and RA vs PsA. The reasons for stopping medication are indicated in 5C. The participants which declared to have stopped medication without been advised by their doctor were asked the reason for not adhering to medications. Reasons are listed as percentages. respondents, bottom female vs male participants.

Non- adherence to medications is a significant problem in the treatment of patients with IA, with many previous studies demonstrating adherence to range from 50-75% ^7, 8, 11^, effects of which impact significantly on patient responses and outcomes. Interestingly, when patients were asked in this study ‘Do you always take your medication exactly as prescribed, other than to be told to stop by your doctor?’ adherence was very strong with >87% *vs* 12% adhering to their medication ( Figure 5A). Adherence to medication was similar amongst female and male participants (87.3% v 87.5%) and also between RA and PsA ( Figure 5A and Table 3). Importantly, 69.9% of those patients who stated that they did not adhere to their medication reported disease flare following cessation of medication ( Figure 5B). The proportion of patients who reported disease flares upon cessation of medication was higher in PsA compared to RA, with no gender differences observed ( Table 3). Of those stopping medication, without the advice of their doctor, the main reasons were ‘feeling better’ (58%), ‘side effects’ (42%) and ‘hard to remember to take’ (39%) ( Figure 5C). A proportion of patients also said that they could not afford their medication and this was their reason for stopping (17%) ( Figure 5C), this represents a significant number of biologic-treated patients ineligible for free medications, who cannot afford them. Altogether, this strongly emphasises the need for patients to strictly adhere to their prescribed medication to maintain disease control. 

### Pregnancy and medication

**Figure 6.**
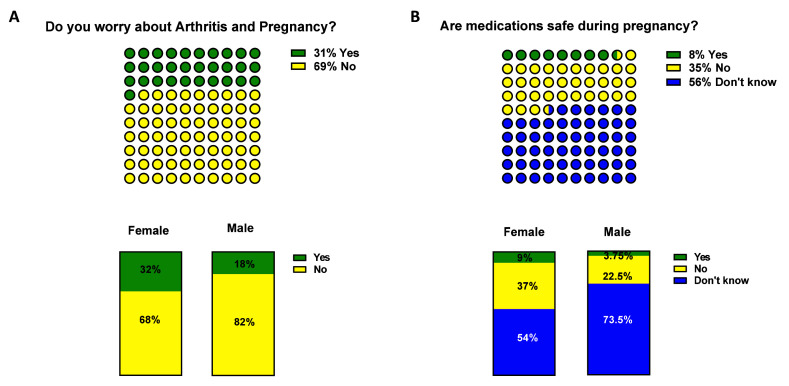
Inflammatory arthritis (IA) and pregnancy. Participants were asked **A**) whether they were worried about arthritis and pregnancy (yellow ‘No’, green ‘Yes’) and **B**) if medication were safe during pregnancy (blue ‘don’t know’, in yellow ‘No’ and green ‘Yes’). Differences among groups are represented in Table 3.

Patients understanding of IA and pregnancy was identified as a potential significant unmet need. Concern that IA would reduce their chances of getting pregnant was reported by 31% of respondents ( Figure 6A). Perhaps of most importance is that the vast majority of respondents either did not know (56%) or believed that arthritis medications were not safe to take during pregnancy (35%) ( Figure 6B). Therefore, only 8% of respondents affirmed knowledge of the safety of arthritis medications during pregnancy, this is a highly significant gap in knowledge in a group of patients who are predominantly female and of childbearing age. Interestingly, a higher proportion of female *vs* male answered definitively ‘No’ regarding the safety of arthritis medication in pregnancy. (37% v 22.5%; Figure 6B), whereas more males answered, ‘did not know’ (73.5% v 54%;) ( Figure 6B and Table 3 p<0.0001). Although, a limitation in the study is that differences in patient education between centres was not captured, this data would highlight there is a need to standardise patient education at all centres nationally.

### Involvement in research

**Figure 7.**
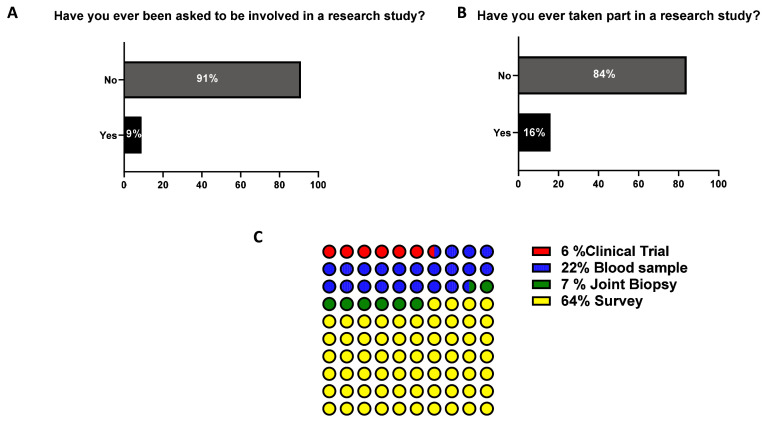
Participants’ involvement in research studies. Participants were asked **A**) whether they were asked in research studies and **B**) whether they took part in a research study. The participants which responded positively to the latter, where asked **C**) which kind of research study they were involved in.

For pre-clinical/translational research to have impact for disease outcomes, patient’s involvement in a meaningful way is critical for its success, thus patient education and engagement is vital. However, in this survey when questioned if patients had ever been asked to be involved in a research study, only 9% of respondents answered Yes ( Figure 7A), with only 16% of these participating in a research study ( Figure 7B). Of those who had taken part in a research study the predominant type of research was a survey (64%), donation of a blood sample (22%), undergoing an arthroscopy (7%), and participating in a clinical trial (6%) ( Figure 7C).

### Participants’ feedback on roundtable workshop discussion

We completed a series of national workshops in which participants were asked to give feedback based on the survey, with an interactive Q&A session between patients and a panel including a rheumatology consultant, a research scientist and a patient partner. Discussion included barriers to patients understanding the various aspects of their disease, from diagnoses and treatment, how to improve medication adherence and suggestions as to what could be incorporated in a research study. Feedback from the workshop was based on the concerns most frequently highlighted in the Q&A session. The effect of anxiety and stress on the disease management and flares were among the highest concerns of patients. These concerns were even more evident in the virtual workshops held during the COVID19 pandemic, where participants voiced their fear of the impact of social isolation, stress, and anxiety on their disease. In addition, participants’ feedback included the role of sleep, diet and exercise on disease management and outcome, and felt there were not enough standardised studies addressing these lifestyle influences on disease management. Finally, the participants felt they were not sufficiently informed on a possible involvement in research studies and clinical trials and felt there were barriers regarding where to find information and defined platforms for engagement in this context. 

## Discussion

The objective of this study was to assess the understanding patients with IA have of their own diagnosis, routine diagnostics tests used in the clinical setting, medication adherence, disease flare, pregnancy and finally to ascertain their attitudes to research studies. The results highlight several patient concerns regarding their disease while also emphasising the need for increased patient education and engagement to improve patients understanding of their disease and develop strong, long-term patient-clinical-researcher partnerships, so that patients can contribute to future project ideas, development, and dissemination.

In this study while a significant proportion of patients understood the difference between IA and OA, a large proportion of respondents expressed concern that IA is a hereditary disease. While there is a genetic component to both RA and PsA, it is now widely accepted that it is a combination of genetic, environmental, and autoimmune factors that contribute to the disease pathogenesis ^12– 15^. Further clarification with patients upon diagnosis regarding these factors and how they can all contribute to the development of IA, may alleviate some concerns about hereditary issues.

A number of tests are utilised in the clinic to aid diagnosis, treatment and follow up of patients with IA. These tests help clinicians to establish a diagnosis but may also be useful to monitor response to treatment and predict disease severity. In this study, we establish that a significant number of patients had little or no understanding of these diagnostic tests, specifically ESR, CRP, RF, ACPA, and the composite disease activity score DAS28. Of note, ACPA was the diagnostic test which patients least understood. Approximately 70% of RA patients test positive for ACPA, with the majority of PsA negative, thus our observation that both RF and ACPA diagnostic test were understood more by RA patients is in line with this. Studies have shown that ACPA ^+^ RA patients develop more erosive disease than ACPA ^-^ patients but they appear to have a better response to therapy ^16^. These tests are pivotal, not alone in making a diagnosis but also understanding the potential course of a patient’s disease, thus enabling clinicians to make decisions regarding treatment. The results of this survey emphasize the need to improve awareness among patients of these possible diagnostic and prognostic tests in order to empower patients to engage in shared decision-making with their rheumatologists. Such patient empowerment is important for patient education, but also towards developing a more meaningful type of patient-clinician relationship whereby patient and doctor can engage in important decision-making as a team rather than as individuals. Several studies examining the impact of patient empowerment on patient outcomes have demonstrated higher levels of patient satisfaction and adherence to treatment regimens, in patients who were actively involved in the decision-making process with their clinician ^17, 18^.

Reassuringly, a significant proportion of respondents in this study indicated that they understood how their medications treat their arthritis and possible side effects that may occur, with <20% of patients unaware of how their medications work or the possible side effects. The development of side effects has been shown to negatively impact adherence to treatment regimens ^19^, therefore, lack of awareness of these potential side effects may lead to increased non-adherence. It is important, therefore that patients are fully aware of the potential side effects of their medications, before they commence treatment, so they can distinguish symptoms associated with side effects and those as a result of the disease itself. Preparedness and increased awareness may reduce anxiety and improve adherence and compliance in their treatment regime. Interestingly, our result suggest that PsA have an overall better understanding of medication side-effect and flaring when medication are stopped, thus suggesting they are more empowered in these aspects.

Interestingly and reassuringly, our patients reported a very high degree of adherence with their prescribed medications compared to those in previous reports in which adherence levels ranged from 50–75% ^7, 8, 10^. It is recognised that non-adherence to DMARD treatment is associated with higher disease activity in early RA patients ^20^. Moreover, a meta-analysis published by Li *et al.,* reporting on 1,963 RA patients demonstrated that DAS28 score was significantly lower in adherent patients compared to non-adherent subjects ^21^. There are far less studies investigating adherence to medication for PsA patients; however, in one retrospective study of 325 patients with PsA, it was reported 76% adherence rate for TNF inhibitors users ^22^. In addition, it was reported that nonadherence to medication was consistently associated with psychological factors in PsA ^23^. These studies suggest that patients who adhere to their medication have better disease control. This is also reflected in our study, whereby >60% of respondents who indicated that they do not always adhere to their treatment regime reported subsequent flare in their disease. Indeed, a previous study by Hill *et al.* reported that an introduction of a patient education programme, involving one to one patient education sessions significantly increased adherence to medication in RA patients ^24^. An increase in patient education regarding flares resulting from cessation of medication, and further clarification on potential side effects may reduce non-adherence further.

 It is well established that the prevalence of RA is significantly higher in females than males where the incidence is 2–3 times higher below the age of 50 ^25^, with PsA prevalence equally distributed between male and female, with males tending to have a higher PASI score ^26^. Consequently, issues regarding pregnancy and childbirth are important considerations for the majority of patients diagnosed with IA, who are females in their reproductive years. This is an issue of particular importance as highlighted in a recent review, following which we established a specific maternal medicine clinic focused patients with arthritis ^27^. In our study, 31% of respondents indicated concern about arthritis and pregnancy while a significant percentage of respondents (91%) also indicated that arthritis medications were not safe in pregnancy, or they did not know. This represents an important unmet need in patient understanding of arthritis medications and pregnancy, which must be addressed by increased education. A study by Chakravarty *et al*., involving two online surveys for physicians and patients regarding family planning issues, reported that the majority of rheumatologists discussed conception and pregnancy with their female patients ^28^. In our experience, patients believed that the information received varied depending on the source and there was little consensus among specialists in relation to the effect of pregnancy on arthritis and of arthritis medication on pregnancy, so we developed a combined update ^29^. In addition, the British Society for Rheumatology has now produced their own guidelines on these issues ^30^. However, less than half reported consulting their patient's treating general practitioner/gynaecologist about these topics. Moreover the majority of patients reported that their pregnancy related concerns were not adequately addressed during their medical appointments ^28^. The results of this survey highlight the safety concerns of medications used to treat arthritis during pregnancy. Rheumatologists should discuss these concerns with patients of childbearing age and address issues such as safety of medication preconception, during pregnancy and during breastfeeding, in addition to highlighting issues regarding disease control before, during and after pregnancy. Rheumatologists should consult the EULAR “points to consider” on the use of anti-rheumatic drugs before and during pregnancy and lactation, when consulting with female patients who are contemplating pregnancy ^31^. Interestingly, as mentioned above, PsA unlike RA which is more common in females, typically affects males and females equally. However, it is important to note that there was a higher proportion of females than males with PsA who participated in our study (5.6:1) which is not reflective of the 1:1 disease dominance which is represented in the PsA general population. Our survey was accessible to everyone, independently from their gender, therefore the higher rate of female participants, are not a reflection of the incidence of the disease, but participation in this study. In our survey, we in fact observed that female have an overall better understanding of their disease, in terms of diagnostic tests and medication efficiency and side effect. Thus, this suggest that female might be more empowered toward their disease understanding than male. We could speculate than female might be addressing more questions during their clinic visit. Previous studies have reported that females experience higher disease activity, higher levels of pain, and lower functional capacity score than men ^32^. Furthermore, females have also documented less favourable patient reported outcomes (PRO) such as pain, fatigue, patient-reported joint count, physical demands at work, work output, and function, compared to males ^33^. Interestingly within our study, there was no significant differences between male and female perceptions to disease understanding, medication and research with the exception that females demonstrated better understanding of how IA medications work than males. This indicates that while PROs and disease burden may be significantly different between females and males, in respect to this study, both genders expressed similar views regarding disease, medications and involvement in research. 

An important aspect of this study assessed the degree of patient involvement in research studies. Our results demonstrate that very few patients have participated in research studies, and this aspect was also highlighted in the Q&A sessions during the national workshops. A small percentage of patients had participated in a study, but the vast majority had taken part in a survey while very few respondents had participated in a clinical trial or bio-sampling studies. Translational research studies are essential in identifying new therapeutic targets for the treatment of IA, improving patient outcomes, predicting response to treatment and predicting disease course. Therefore, patient participation in these studies is critical in helping to achieve these aims. Through the patient centred workshops completed during this study, it is clear that many patients are willing and keen to take part in such studies but need more information as to how and when to engage with the research team in this respect, and this engagement needs to take place at the development stage of the research. Indeed, patients highlighted the need for studies that address the link between lifestyle and level of inflammation, with a particular emphasis on sleep, diet, anxiety, and exercise. Therefore, we need a structured environment for researchers (clinical and scientific) and patients to interact at the pre-clinical stage of research studies to enable patient input. Furthermore, it is important that principal investigators actively recruiting patients for studies disseminate their interest to a wide variety of patient groups in clinical settings outside hospitals, such as GP surgeries, advocacy groups and community health centres.

To the best of our knowledge, this is the first study to examine and capture patient perceptions and disease understanding in both RA and PsA patients in the Irish community. However previous work by de Jong et al, demonstrated that in order to involve patients in their own care and improve patient education, a questionnaire assessing the education “gaps” would be effective in designing education tools ^34^. Subsequently the group have developed an item bank for measuring patient knowledge in RA, which can be used by health professionals and researchers to identify and target patients’ educational needs and thus represents an additional tool to address some of the educational gaps identified within our study ^34^.

Finally, we recognise specific limitations within this study, most notably the design of our online survey which failed to capture more in depth clinical and socio-economic detail. It is widely accepted that response rates to surveys are directly correlated with survey length, with longer surveys having reduced participation. We therefore chose to focus on key questions related to understanding of disease, medication disease flare, heredity, pregnancy, and patient involvement in research, which fell within the scope of this study. However, it would also be of high interest and indeed add greater value to our conclusions to understand the socio-economic background of the participants and whether this has an effect on the parameters measured in this study. However, while we did not measure socioeconomic factors directly, it is interesting to note that when participants who stopped taking their medications without seeking medical approval were asked why, 17% responded that they could not afford them. This is suggestive that socioeconomic factors may indeed paly an important role in IA patient experience and understanding of disease. Furthermore, and additional limitation, which was outside the scope of this study was assessing the disease duration of the study participants which may also have an effect on disease understanding and patient involvement in research. Future work should aim to assess the patients’ perspective of their disease in those newly diagnosed compared to those with long standing disease and most notably how that perspective may change as their disease progresses. Importantly, our study also considers both RA and PsA patients and their perceptions which understandably may be considerably different given the additional burden of skin disease seen in PsA patients. Our data, in fact, highlight a different understanding in side effect and possibility in flaring after stopping medication between RA and PsA patients, with the latter displaying a better understanding of these factors. However, PsA patients worry more about the possibility that their arthritis might have an effect on pregnancy, thus suggesting that these patients are less informed on this aspect. In conclusion, this study analyses the patient’s perspective of their disease, and the challenges and concerns they have with regard to their diagnoses. We used an approach of surveying patients and organizing educational workshops to ascertain and fill the knowledge gaps and thus engage with a wide patient audience. This unique collaborative approach involved rheumatologists, scientists, a patient partner, and the national patient advocate group - Arthritis Ireland to identify conceptually important and relevant constructs to develop the ‘patients awareness survey’, specifically addressing items of understanding of diagnosis, diagnostic tests, medication and adherence, disease flare, heredity, pregnancy and patient involvement in research. This survey was designed with the scope to investigate patient’s understanding of their disease; therefore, we did not add physician perspective in this study. This survey facilitated an innovative approach to engage patients through the development of a national network of ‘patient education and awareness workshops’, with meaningful engagement with the patients, the clinicians and the scientists. The results are informative, positively highlighting patients have a good understanding of their medication and adherence and are eager to engage in research studies. Importantly however, we also identified significant ‘gaps’ in patient education, which highlighted poor understanding of diagnostic tests, concerns with regard to pregnancy and hereditary and lack of engagement in the development of research studies, these are important knowledge gaps that we must addressed in our practice plan. Importantly one tool which may help both clinicians and patients more efficiently monitor disease status, is the use of health related digital app’s, which have been highly successful, for instance, during the COVID19 pandemic, to improve public health governance ^35^, however a caveat of these platforms is that clinicians may still be reluctant on the use of these apps, displaying reservations and uncertainties regarding their expertise in interpreting results, as well as privacy and transparency issues.

## Consent

Informed consent for publication of the participants survey answers was obtained from the participants at the time of survey completion.

## Data Availability

Repository: FigShare. IA Survey Spreadsheet Data PPI April 2021 DOI: 10.6084/m9.figshare.14414144 This project contains the following underlying data IA Survey Spreadsheet Data PPI April 2021 (Responses to online survey from RA and PsA patients assessing their understanding of their disease, diagnostic tests, medication, pregnancy, and involvement in research) Data are available under the terms of the Creative Commons Zero "No rights reserved" data waiver (CC) 1.0 Public domain dedication)
