# Supplementary material for: Knowledge of disease, diagnosis, adherence and impact of research in an Irish cohort of patients with inflammatory arthritis
Source: HRB Open Res. 2023 Apr 27;4:60. Originally published 2021 May 28. [Version 2] doi: 10.12688/hrbopenres.13274.2 (PMC10213824; doi:10.12688/hrbopenres.13274.2)
Supplement: Peer review history

# Peer review: Reviewer response for version 2

**DOI:** 10.21956/hrbopenres.14973.r33688

**Published:** 2023-05-25

**Authors:** Senem Sas

**Affiliations:** 1 Faculty of Medicine, Department of Physical Medicine and Rehabilitation, Division of Rheumatology, Erciyes University, Kayseri, Turkey

The manuscript can be indexed. Regards

Is the work clearly and accurately presented and does it cite the current literature?

Yes

If applicable, is the statistical analysis and its interpretation appropriate?

Yes

Are all the source data underlying the results available to ensure full reproducibility?

Yes

Is the study design appropriate and is the work technically sound?

Yes

Are the conclusions drawn adequately supported by the results?

Yes

Are sufficient details of methods and analysis provided to allow replication by others?

Yes

Reviewer Expertise:

Rheumatology

I confirm that I have read this submission and believe that I have an appropriate level of expertise to confirm that it is of an acceptable scientific standard.

# Peer review: Reviewer response for version 2

**DOI:** 10.21956/hrbopenres.14973.r33687

**Published:** 2023-05-03

**Authors:** Ruben Queiro

**Affiliations:** 1 Rheumatology Division, Hospital Universitario Central de Asturias (HUCA), Oviedo, and ISPA Translational Immunology Division, Oviedo University School of Medicine, Oviedo, Spain

For my part there is nothing more to suggest.

Is the work clearly and accurately presented and does it cite the current literature?

Yes

If applicable, is the statistical analysis and its interpretation appropriate?

Partly

Are all the source data underlying the results available to ensure full reproducibility?

Partly

Is the study design appropriate and is the work technically sound?

Yes

Are the conclusions drawn adequately supported by the results?

Yes

Are sufficient details of methods and analysis provided to allow replication by others?

Partly

Reviewer Expertise:

Rheumatic diseases, spondyloarthritis, psoriatic arthritis, outcome measures.

I confirm that I have read this submission and believe that I have an appropriate level of expertise to confirm that it is of an acceptable scientific standard.

# Peer review: Reviewer response for version 1

**DOI:** 10.21956/hrbopenres.14447.r32420

**Published:** 2022-08-30

**Authors:** Senem Sas

**Affiliations:** 1 Faculty of Medicine, Department of Physical Medicine and Rehabilitation, Division of Rheumatology, Erciyes University, Kayseri, Turkey

A well-designed and written study. An online anonymous survey was distributed to patients living with inflammatory arthritis which addressed questions about diagnosis, routine tests, medications and how they work, medication adherence, disease flare, heredity, pregnancy, and patient involvement in research. The study suggests stronger relationship with patient and physician.

 Material methods must be more detailed. A professional statistical consultant is needed to analyze the data. The study is heterogeneous. The disease activity scores are calculated in different ways. Logistic regression analysis can be done. Why were the associations of education level and knowledge of disease and disease activity not studied?

 Discussion: genetic factors are unnecessary. The study is patient focused questionnaire, so I suggest shorten the genetic factors. Authors can summarize it in one sentence. The discussion should be rewritten in the light of the study findings. The findings of the study should be emphasized. The study limitations must be mentioned. The limitations; the study group is heterogeneous, the education level is absent and the physician perspective is absent.

 In conclusion: The authors may add awareness of social media among physicians.

Is the work clearly and accurately presented and does it cite the current literature?

Yes

If applicable, is the statistical analysis and its interpretation appropriate?

Yes

Are all the source data underlying the results available to ensure full reproducibility?

Yes

Is the study design appropriate and is the work technically sound?

Yes

Are the conclusions drawn adequately supported by the results?

Yes

Are sufficient details of methods and analysis provided to allow replication by others?

Yes

Reviewer Expertise:

Rheumatology

I confirm that I have read this submission and believe that I have an appropriate level of expertise to confirm that it is of an acceptable scientific standard, however I have significant reservations, as outlined above.

# Peer review: Reviewer response for version 1

**DOI:** 10.21956/hrbopenres.14447.r31898

**Published:** 2022-05-04

**Authors:** Ruben Queiro

**Affiliations:** 1 Rheumatology Division, Hospital Universitario Central de Asturias (HUCA), Oviedo, and ISPA Translational Immunology Division, Oviedo University School of Medicine, Oviedo, Spain

This interview with Irish patients affected by chronic inflammatory arthritis sheds light on a series of aspects that are key to planning quality care for this group of patients. For the purposes of the study, an online anonymous survey was distributed to patients living with inflammatory arthritis (RA and PsA) which addressed questions about diagnosis, routine tests, medications and how they work, medication adherence, disease flare, heredity, pregnancy, and patient involvement in research. The study concludes on the need to create strong links between patients and health care providers, to involve patients more and better in decision-making and in research projects. Overall the study is interesting and noteworthy.

 Aspects to consider in order to improve the final manuscript:

- A greater in-depth study of the socio-demographic characteristics of the patients surveyed is lacking. For example, nothing is provided about the sociocultural level (level of studies, urban or rural origin, employment situation, for example) of the participants; neither are we told anything if they are recently diagnosed patients, or if they are patients with long-standing disease. The latter is important, since the experience of the disease and the knowledge related to it is not the same in patients with different times of disease evolution.
- It seems that the authors include diagnostic tests and disease outcomes such as DAS28 in the same section. I think this should be treated differently. On the other hand, the DAS28 is an assessment tool more typical of RA than of PsA. This should be mentioned in the discussion.
- There is a clear representation of the female gender over the male. This can be understood in RA, but not so much in PsA. The way men and women experience their illness is different, and this bias should be mentioned in the discussion.
- In the statistical methodology it is said that the chi-square statistic is used, and no other details are mentioned. How were the quantitative variables analyzed (the chi-square is not applicable here)? If comparisons of 3 or more variables were made, what statistic was used? Was the sample selection random, and in that case, how was it done? Is the sample representative of the Irish population with these pathologies?
- In the discussion, please mention similar works to yours and in which they coincide or differ with respect to your results, if it is the case. Mention the pros and cons of your survey. Above all, address the potential biases that come with a survey like yours. Perhaps a weak point of the survey was mixing patients with two very different diseases (keep in mind that PsA patients actually experience two illnesses in one). It would have been more conclusive to treat both diseases separately with an adequate number of patients in each study arm.

Overall, the study is well done and the information it provides should be disseminated.

Is the work clearly and accurately presented and does it cite the current literature?

Yes

If applicable, is the statistical analysis and its interpretation appropriate?

Partly

Are all the source data underlying the results available to ensure full reproducibility?

Partly

Is the study design appropriate and is the work technically sound?

Yes

Are the conclusions drawn adequately supported by the results?

Yes

Are sufficient details of methods and analysis provided to allow replication by others?

Partly

Reviewer Expertise:

Rheumatic diseases, spondyloarthritis, psoriatic arthritis, outcome measures.

I confirm that I have read this submission and believe that I have an appropriate level of expertise to confirm that it is of an acceptable scientific standard, however I have significant reservations, as outlined above.
